# Department of Therapeutics

**Published:** 1894-02

**Authors:** 

**Affiliations:** Demonstrator of Physiology and Lecturer on the History of Medicine in the Medical Department of the University of Texas, Etc.; Galveston


For Texas Medical Journal.

### DEPARTMENT OF THERAPEUTICS.

EDITED BY DAVID CERNA, M. D., PH. D.,
Demonstrator of Physiology and Lecturer on the History of Medicine in the Medical Department of the University of Texas, etc.

THE THERAPEUTIC USES OF EXALGIN.—E. G. Porter (*Bull. Génér. de Thérapeutique-Therapeutic Gazette*, December 15, 1893) reports three cases in which exalgin proved serviceable in the treatment of neuralgic pain of functional nature. These good results led the observer to try exalgin in cases of mental disease, associated with subjective symptoms similar to those of neuralgic disorders. He details five cases of mental disease in which the drug gave highly satisfactory results. The use of the remedy in these cases was mainly empirical, the author offering no explanation as to the manner in which exalgin acts. He employed the medicament particularly in those hopeless cases characterized by melancholia, accompanied with marked headache and insomnia. The mental state was not ameliorated, but in every case, however, exalgin relieved the two latter symptoms,—that is, the headache and the insomnia. The drug never produced untoward effects, although the writer never gave it in larger doses than two grains every four hours. Generally he administered it in repeated doses of one grain each, a method that was sufficient to produce the desired effect.

---

A New Remedy in the Treatment of Epilepsy.—At a recent meeting of the Medical Society of the county of New York, Paul Gibier presented a second contribution to the treatment of epilepsy by injections of a nervous (cerebral) extract, his first paper having been read about a year ago. Since then he had continued his observations upon the old cases, and had added new ones. The new ones, which had been markedly improved under the new agent, were about nine in nnmber, of which he read a brief history. Four others, which had been submitted to the treatment, had not done any better than they had formerly done under bromides, and therefore the treatment was discontinued. Of the older cases he continued the history of two, both of which had been under treatment about eighteen months, the improvement, very striking in one, having persisted as long as the injections were kept up. Most of the cases gave a history of grand mal existing several years or from infancy, and in all, excepting the four already mentioned, the improvement had been marked, the grand mal, as a rule, having been converted into petit mal, the attacks appearing at longer intervals, the bodily and mental condition also showing improvement. Regarding the mode of action of the remedy, Gibier believes that, theoretically, one might suppose the nervous substance, which was injected, supplied a want in the nervous centres from which the epileptic attacks started, and enabled them to resist the discharge. Of course, as long as we remained ignorant of the nature of essential epilepsy, any explanation of a remedy would be only hypothetical.—*Medical Record, January 13, 1894.*

---

The Treatment of Convulsive Attacks by Anæsthetics.—In the *Medical Record* or October 31, 1893, W. S. Magill described what he termed a "new and rapid method of anæsthesia," which consists principally in the administration of ethyl bromide first, this being continued by that of chloroform. In a later note (*Medical Record*, January 13, 1894) Magill invites attention to the utility of this combined process of anæsthesia for the abortive treatment of convulsive attacks in eclampsia, epilepsy and hysteria. The rapid action of the ethyl bromide-abolition of reflex action in thirty to sixty seconds, serves frequently to abort completely the oonvulsion; and in case of persistence of the tendency to convulsions (subintrans), the continuance of anæsthesia with chloroform prolongs the therapeutical action *ad lib.* Theoretically the stimulus of the salivary and sudoral glands (organs of elimination), exercised by the inhalation of ethyl bromide, should be particularly useful in eclampsia and other convulsions of toxic origin.

---

Cocaine Wakefulness.—An interesting case of wakefulness by cocaine is reported by J. W. Stickler (*Medical Record*, January 13, 1894). The awakening effect of cocaine was not overcome by comparatively large doses of chloral and opium, as will be seen in the following instance: For the pain of toothache a patient took into his mouth nine grains of cocaine in solution, a small portion at a time, holding it till the accumulation of salvia became so abundant that he had to spit it out. He began using the cocaine in this manner at 5 p. m., and did not cease till 10.22 p. m. the same evening. As it was then bed time he thought he would make sleep certain by taking twenty grains of chloral. Immediately after taking the latter drug he took into his mouth some more cocaine and went to bed. He "swashed" the cocaine solution about in his mouth awhile, then spat it out, turned on his side and tried to go to sleep. Sleep, however, did not come; on the contrary, he did not even become drowsy. Having lain awake till midnight, and not feeling sleepy at that hour, he took, as nearly as he could tell, about *one teaspoonful* of laudanum. He went to bed again and remained awake till 3 o'clock. Sleep lasted only two hours. Following this there was headache.

---

Brown Sequard's Fluid in Skin Affections.—Monnet (quoted by Brocq in *Jour. of Cutan. and Gener. Urin. Dis.*) has tried injections of testicular juice in a young girl of nervous temperament, affected with symmetrical leucodermia of the trunk and lower extremities. Three cubic centimeters were injected morning and evening for six weeks. The general condition improved and the discoloration became gradually less, and at the end of three months the disease had almost entirely disappeared.

Monnet has tried the same method in other skin affections, including three cases of ichthyosis, fourteen of neurotic eczema, several of "trophoneurotic erythema," and several of bullous and vesicular eruptions in the subjects of hemiplegia and general paralysis. Monnet explains the effect of the remedy in cutaneous trophoneurosis by its action on the nervous system, which is the source of the trouble. He thinks that injections of cerebrine would be more effectual in these cases. Brocq, while carefully avoiding committing himself on the subject, "cannot deny that the ideas on which rest the attempts of Monnet are both logical and perfectly rational.

---

TUBERCULIN TREATMENT IN EGYPT.—According to the *Brit. Med. Journal*, Schiess and Kartulis give results of treatment with tuberculin in forty-eight tuberculous patients. They find that in the Egyptian climate the treatment is harmless if commenced with small doses, and that even patients with advanced phthisis may be treated by this method. They have compared their cases with others in which, though tuberculin was not used, the other conditions were the same. Their conclusions are in favor of the use of tuberculin; by its aid, they say, commencing pulmonary tuberculosis gets well certainly, and in a few months, while advanced cases may also recover, although more slowly. Very severe cases, with vomicæ, hectic fever and night sweats, are unsuitable for this treatment. Scrofuloderma got well more quickly than lupus, and tuberculin was also found useful in certain tuberculous affections of the joints and bones, in combination with surgical treatment. The Egyptian climate is, they think, especially suitable for the tuberculin treatment.

---

BORIC ACID IN TYPHOID FEVER.—The *Union Mêdicale* for November 7th gives a resumé of an article by Dr. L. Tortchinsky, published in the *Gazette Hebdomadaire de Bordeaux*. The author used boric acid in two hundred and forty cases of typhoid fever in the course of an epidemic, and reports excellent results; only nine patients died, and they succumbed during the period of convalescence because they got out too soon or committed errors in diet. The two hundred and thirty-one other patients made a rapid and complete recovery. In all the cases the patients were given a dose of castor oil with from five to ten drops of oil of turpentine. After this mixture had operated the administration of boric acid was begun, the remedy being given internally, either in powder or in solution, in doses ranging from twelve to fifteen grains for an adult three or four times a day. When there was bronchitis the boric acid was associated with expectorants and with hydrochloric acid. As a general rule, at the end of from three to five days the fever and the diarrhœa underwent a noteworthy diminution, the tympanities disappeared, the dejecta lost their odor and became normal in appearance, the urine became abundant and in every way normal, the tongue and skin grew moist and the general condition was good. As soon as the amelioration was well marked the use of the acid was discontinued and tonics were ordered. Under the influence of this treatment the disease followed a favorable course, its duration was somewhat diminished, and complications were very rare. The most decided effects were obtained in cases treated early. The author has found that the effects of the boric acid treatment may be increased by combining with that drug small doses of acetanilide, quinine, naphtaline, or salol. The mixture with quinine is especially useful in the stages of the fever, when there are ataxia, delirium, and other cerebral symptoms; it is useful, also, in cases of relapse. The author has never observed any harmful effect from the use of boric acid. He has also produced satisfactory results with this acid in the treatment of the summer diarrhœa of children.—*N. Y. Med. Journal*, Dec. 16, 1893.

